# Supplementary figures and images for: Exploration into Natural Variation Genes Associated with Determinate and Capitulum-like Inflorescence in Brassica napus
Source: Int J Mol Sci. 2023 Aug 17;24(16):12902. doi: 10.3390/ijms241612902 (PMC10454214; doi:10.3390/ijms241612902)

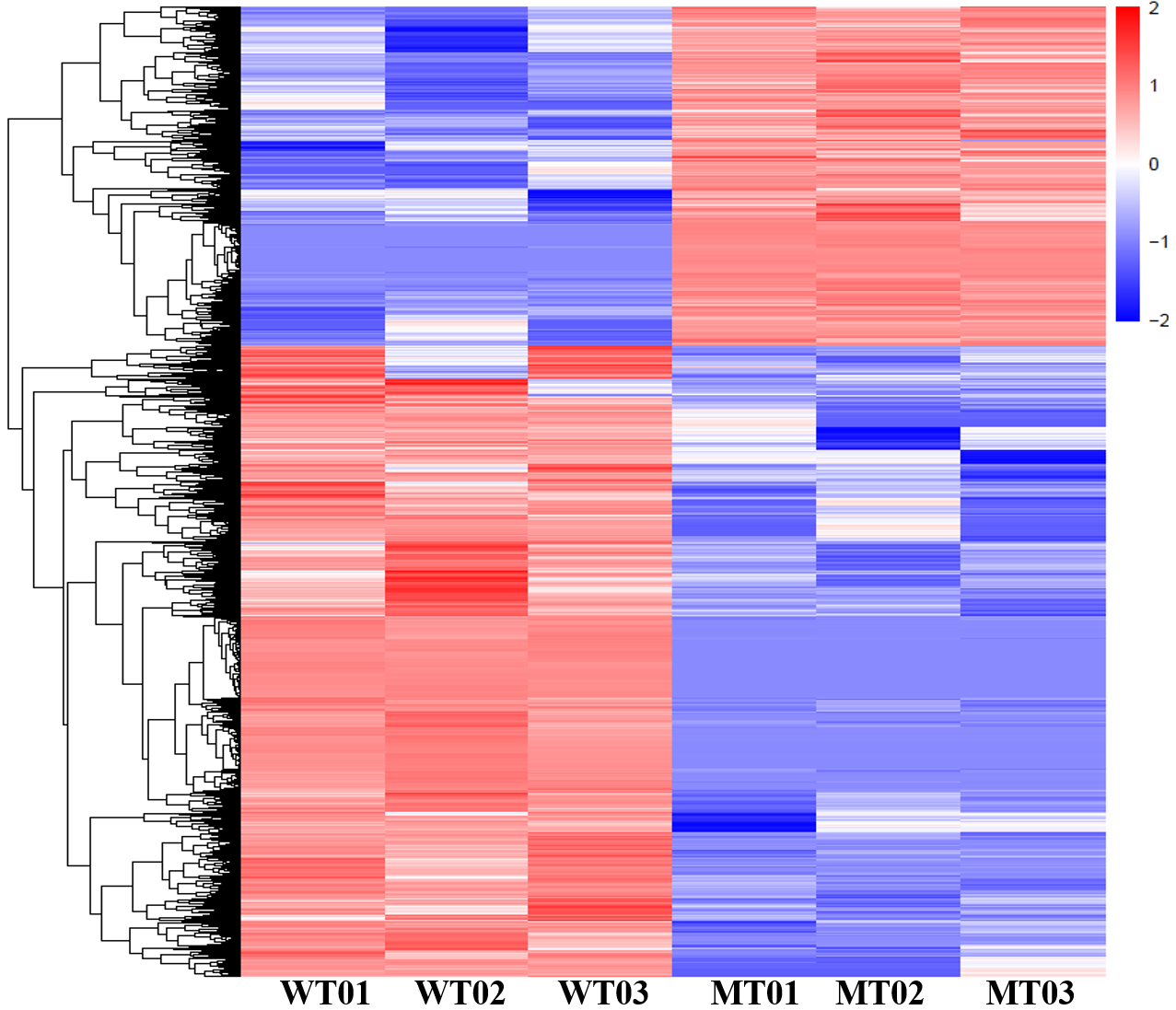

Supplement: Supplementary file 1 [file ijms-24-12902-s001.zip › Figure S1.tif]

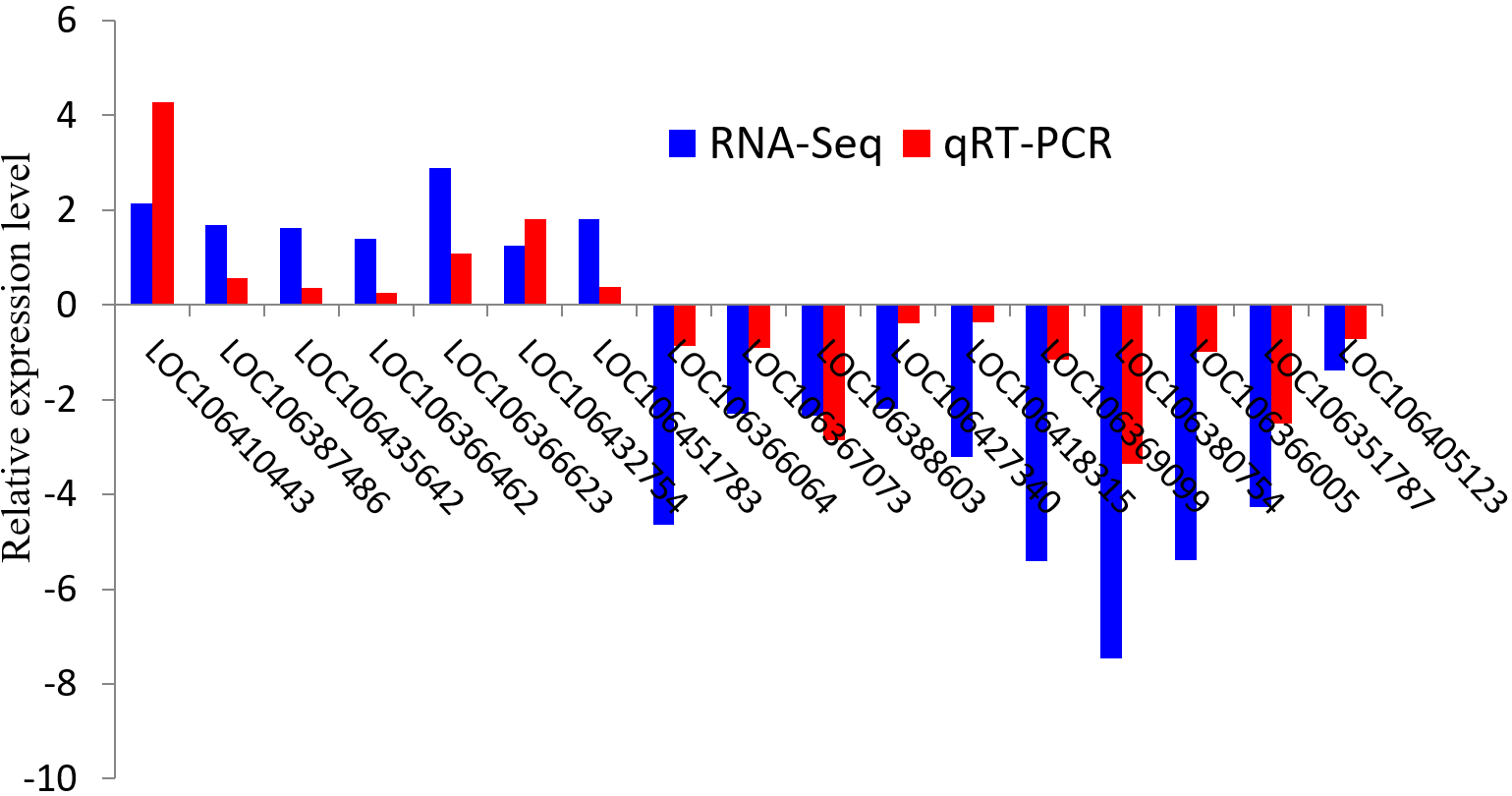

Supplement: Supplementary file 1 [file ijms-24-12902-s001.zip › Figure S2.tif]

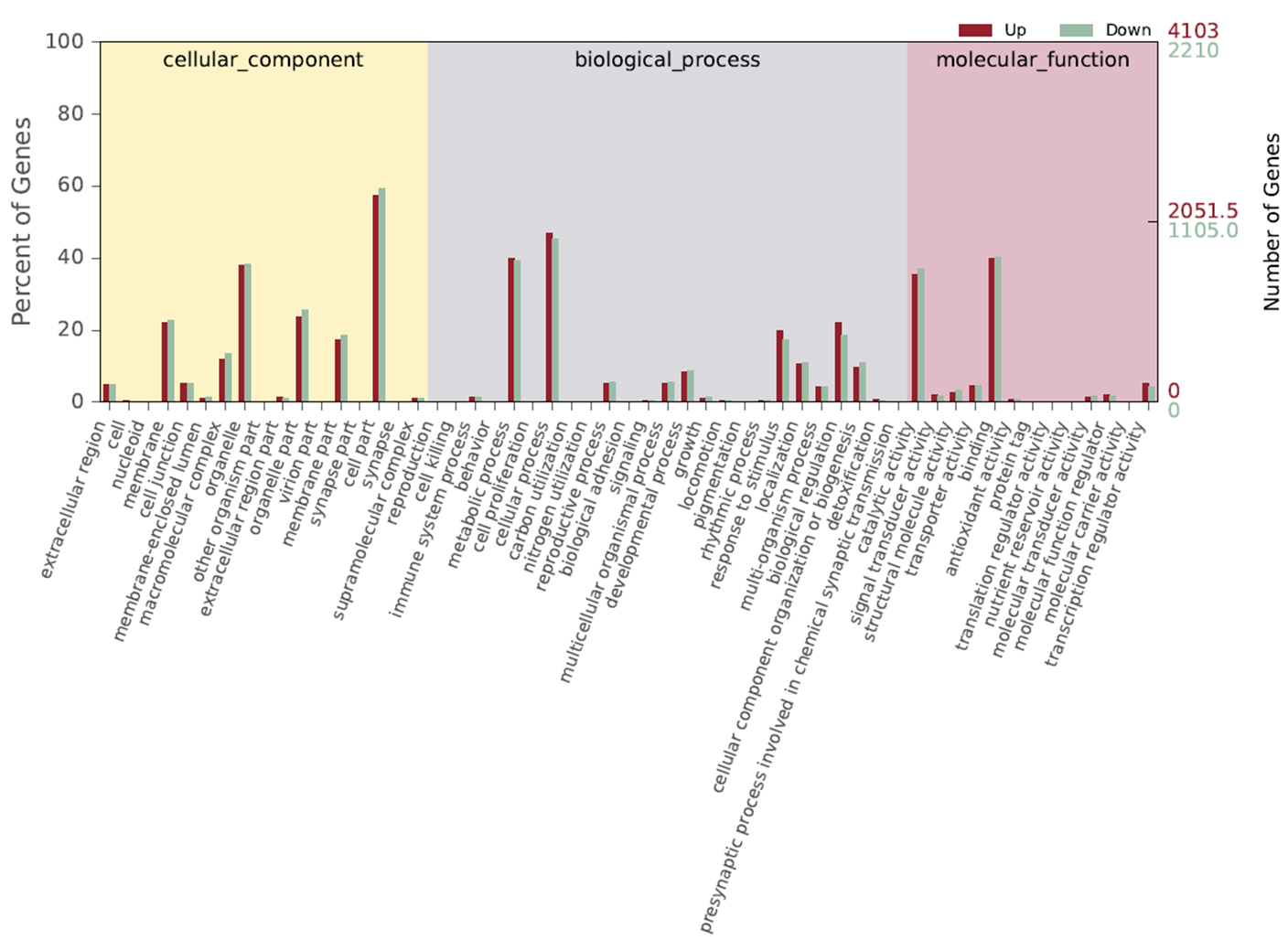

Supplement: Supplementary file 1 [file ijms-24-12902-s001.zip › Figure S3.tif]

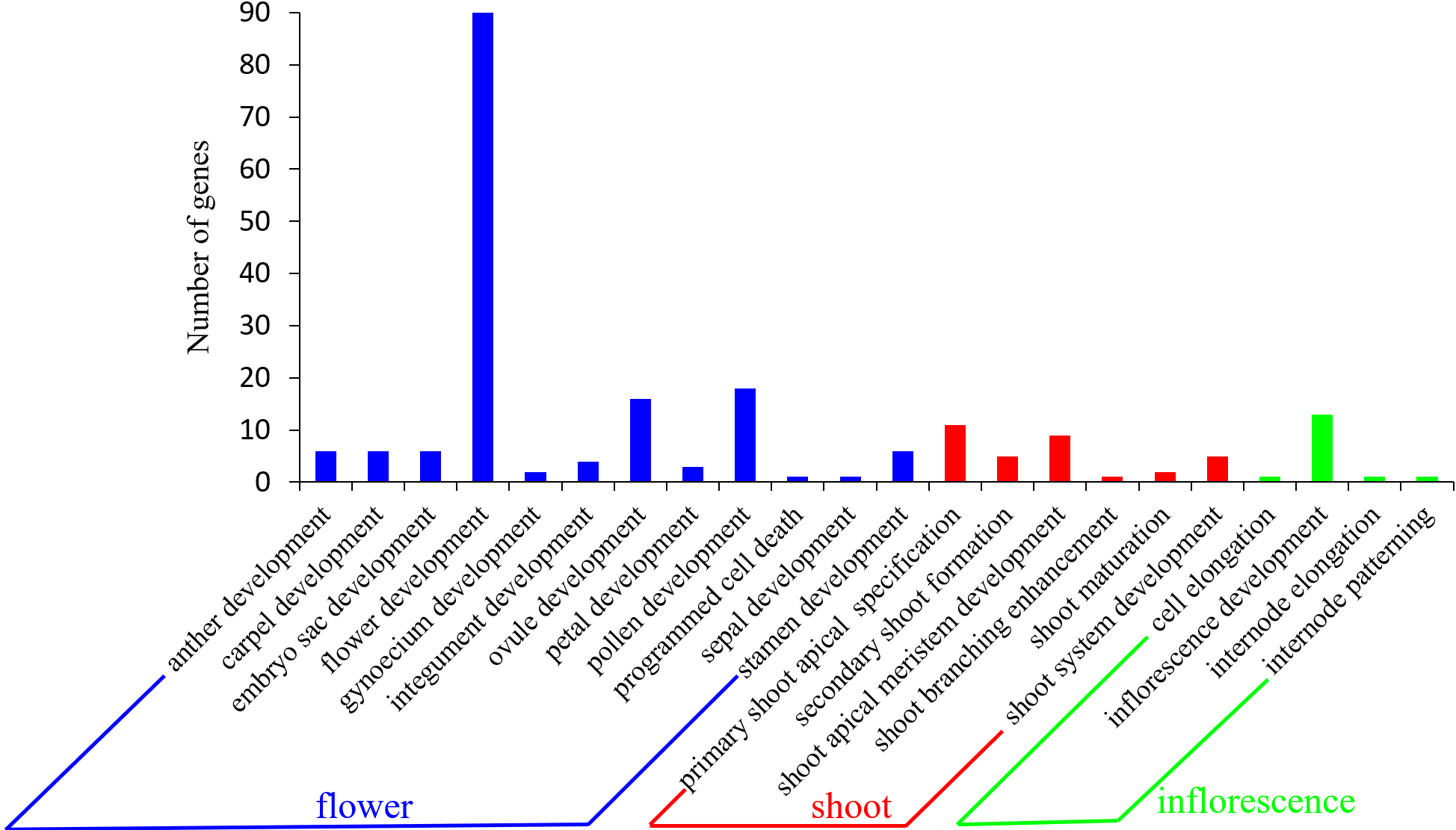

Supplement: Supplementary file 1 [file ijms-24-12902-s001.zip › Figure S4.tif]
